# Critical assessment of chromatographic metadata in publicly available metabolomics data repositories

**DOI:** 10.1007/s11306-022-01956-x

**Published:** 2022-11-27

**Authors:** Eva-Maria Harrieder, Fleming Kretschmer, Warwick Dunn, Sebastian Böcker, Michael Witting

**Affiliations:** 1 Research Unit Analytical BioGeoChemistry, Helmholtz Zentrum München, Ingolstädter Landstraße 1, 85764 Neuherberg, Germany; 2 Chair of Bioinformatics, Friedrich-Schiller-Universität Jena, Ernst-Abbe-Platz 2, 07743 Jena, Germany; 3 Department of Biochemistry and Systems Biology, Institute of Systems, Molecular, and Integrative Biology, University of Liverpool, Liverpool, L69 7ZB UK; 4 Metabolomics and Proteomics Core, Helmholtz Zentrum München, Ingolstädter Landstraße 1, 85764 Neuherberg, Germany; 5 Chair of Analytical Food Chemistry, TUM School of Life Sciences, Technical University of Munich, Maximus-Von-Imhof-Forum 2, 85354 Freising, Germany

**Keywords:** Data reuse, Retention time, Repositories, Metabolomics, LC-MS

## Abstract

**Introduction:**

The structural identification of metabolites represents one of the current bottlenecks in non-targeted liquid chromatography-mass spectrometry (LC–MS) based metabolomics. The Metabolomics Standard Initiative has developed a multilevel system to report confidence in metabolite identification, which involves the use of MS, MS/MS and orthogonal data. Limitations due to similar or same fragmentation pattern (e.g. isomeric compounds) can be overcome by the additional orthogonal information of the retention time (RT), since it is a system property that is different for each chromatographic setup.

**Objectives:**

In contrast to MS data, sharing of RT data is not as widespread. The quality of data and its (re-)useability depend very much on the quality of the metadata. We aimed to evaluate the coverage and quality of this metadata from public metabolomics repositories.

**Methods:**

We acquired an overview on the current reporting of chromatographic separation conditions. For this purpose, we defined the following information as important details that have to be provided: column name and dimension, flow rate, temperature, composition of eluents and gradient.

**Results:**

We found that 70% of descriptions of the chromatographic setups are incomplete (according to our definition) and an additional 10% of the descriptions contained ambiguous and/or incorrect information. Accordingly, only about 20% of the descriptions allow further (re-)use of the data, e.g. for RT prediction. Therefore, we have started to develop a unified and standardized notation for chromatographic metadata with detailed and specific description of eluents, columns and gradients.

**Conclusion:**

Reporting of chromatographic metadata is currently not unified. Our recommended suggestions for metadata reporting will enable more standardization and automatization in future reporting.

**Supplementary Information:**

The online version contains supplementary material available at 10.1007/s11306-022-01956-x.

## Introduction

The identification of metabolites is one of the major bottlenecks in non-targeted metabolomics. Current identification strategies strongly rely on the use of high-resolution mass spectrometry (MS) and high-resolution MS/MS data, as well as nuclear magnetic resonance spectroscopy for structure elucidation. However, the latter requires larger amounts of ideally pure substances for detailed structural analysis, which is often not available. Therefore, different approaches have been developed to derive as much structural information as possible from MS and MS/MS data. A typical first step is by matching tandem MS spectra of unknown metabolites against reference spectra from different in-house or external mass spectral databases. Various publicly available tandem MS databases exist, e.g. Metlin, MassBank, MassBank of North America, Global Natural Products Social Molecular Networking (GNPS) and others (MassBank Europe, 2022; MassBank of North America (MoNA), 2022; Schulze et al., 2021; Smith et al., 2005; Wang et al., 2016; Xue et al., 2020). However, the number of spectra is often limited by the number of commercially available reference standards. Therefore, novel approaches have been developed to search with tandem MS data in chemical databases, which are typically several orders larger than tandem MS spectra databases. These approaches include a vast array of different in silico tools, e.g. derivation of a fingerprint from the molecular structure or predicting the fragmentation tree based on tandem MS data (Dührkop et al., 2015, 2019; Ridder et al., 2012; Ruttkies et al., 2016; Tsugawa et al., 2016).

However, MS and tandem MS data typically fail at the identification of molecules with very close or almost similar structures (e.g. isomeric metabolites). Orthogonal information such as retention time (RT) from chromatographic separation or collisional cross sections from ion mobility separation can be used to further narrow down the list of potential candidates and strengthen the additional confidence in the identification. Nevertheless, RT is often neglected in the initial stages of metabolite identification and only used for comparison against reference standards, despite being represents valuable orthogonal information on metabolite polarity (polar metabolites can be better separated with hydrophilic interaction chromatography, whereas reversed phase (RP) chromatography better suits non-polar metabolites). In contrast to MS and MS/MS data, which represent mainly structural properties (with some dependency on fragmentation type, instrument and energies), RT can be described as a system property, since it depends on the analyte of interest, the employed chromatographic column, the solvents and many other experimental parameters. This means that there is not “the one” RT of a metabolite, but it varies depending on the applied chromatographic system, which complicates a comparison of two different chromatographic systems, even when using nominally the same chromatographic setup (meaning the same column, eluent and gradient). In Gas Chromatography (GC) normalization of RTs can be achieved by conversion into retention indices (RI). A commonly used method is Kováts RI, where a series of n-alkanes is used as reference standards for nominalization (Kováts, 1958). This is widely used on GC for the identification of molecules in metabolomics. Different approaches have been developed for the use of RI in LC (Stoffel et al., 2022; Zheng et al., 2018).

RT prediction represents an interesting new tool for metabolite identification by helping to reduce the number of potential candidates already reported in an early stage of the metabolite identification workflow using MS data, but requires high-quality training data for development, including sufficient information on the chromatographic system and assay (Witting & Böcker, 2020). While the collection of MS and MS/MS data in repositories is widespread and considered to be necessary, the collection and storage of RTs is often neglected. Typically, RTs are stored in in-house databases along with MS/MS data. It is often believed that RTs cannot be (re-)used to a similar extent as MS and MS/MS due to being restricted to a specific system, but this is only partially true. A particular example for sharing and (re-)use of RT data is PredRet, which uses commonly detected metabolites between similar chromatographic systems to perform projections between them (Stanstrup et al., 2015). In another approach, RT data of the same biological samples were first mapped between different instruments and then combined to increase the coverage of detected metabolites (Vaughan et al., 2012).

Similar to MS and MS/MS data, RT information requires extensive collection of metadata of the applied experimental parameters to be useful for metabolite identification, since it heavily depends on the nature of the chromatographic setup. While for MS experiments, metadata is normally (but not always) collected and reported, details of chromatographic separations are only partially reported and at different levels of detail.

In 2005, the Metabolomics Standards Initiative (MSI) aimed to identify, develop and propose a common description for best chemical analysis practice in metabolomics (Sumner et al., 2007). This includes the formulation of a minimal set of reporting standards that describe the experimental methods that are necessary to make data accessible and (re-)usable for other researchers. One part of this is the definition of a minimal set of metadata of the chromatographic section that should be specified. This information includes a description of the chromatographic instrument (e.g. name, manufacturer, software package), the auto-injector (e.g. type, software version, injection volume), the (guard-)column (e.g. manufacturer, name, stationary phase, physical parameters), the technique-specific sample preparation (e.g. derivatization, spiking, resuspension) and the separation parameters (e.g. method, injector temperature, mobile phase composition, flow rate).

While searching for publicly available RT datasets in different repositories, we realized that this minimal information is often not or only partially present in chromatographic descriptions. That is also in line what the observation Spicer et al. made in 2017, who conducted a study evaluating the compliance of the datasets, focusing on biological context, in four repositories that should follow and fulfill the MSI guidelines for minimal reporting standards (Spicer et al., 2017).

In order to investigate how extensively such metadata is reported and whether it is sufficient for the (re-)use of RT data in metabolite identification workflows, we analyzed the protocol and chromatography sections of the two publicly available repositories MetaboLights (housed at EMBL-EBI in the UK) and the Metabolomics Workbench (housed at the University of California San Diego in the USA) and collected the metadata of studies which used liquid chromatography-MS (LC–MS) based workflows (Haug et al., 2019; Sud et al., 2016). We decided to use data from these two repositories since they represent a cross-section of studies from different fields in which metabolomics is applied. Since protocol sections are typically copied from the corresponding material and methods section of the published papers, they also represent the current practice of reporting chromatographic separation methods in scientific papers. We extracted information on the column name, length, inner diameter, particle size, flow rate, temperature, composition of eluents and the applied gradient. We assumed that data deposited in these two databases represent a good approximation for the universe of all metabolomics studies.

## Material and methods

### EMBL-EBI MetaboLights and NIH Metabolomics Workbench datasets

We manually collected data and chromatographic conditions from publicly available MetaboLights and Metabolomics Workbench studies using LC–MS (full list in SI Table 1, as of 2020/10/01). We extracted the following information from the experimental descriptions: column name, length, inner diameter, particle size, flow rate, temperature, composition of eluents and gradient. Missing information was marked as “NA”. Statistical analysis was performed with R (version 4.1.2) in RStudio. In the case of column names, a potential matching to standardized column names (see below) was performed manually.

### Collection of standardized column names, solvents, and additives

Column names and additional information were extracted from the websites, brochures and catalogs of different column vendors (e.g. Agilent, Waters, Thermo Fisher, Dr. Maisch, Phenomenex and others). The curated data was collected into a central spreadsheet; standardized column names and additional information, e.g. on the USP code (code of grouping the columns, developed by the United States Pharmacopeia (USP) convention, based on the phase material, e.g. C_18_ are L1, C_8_ are L7, phenyl-phases are L11), particle size, column inner diameter, length, particle size and pore size for a large portion of curated columns, were included. Column naming includes the correct brand naming obtained from the information supplied by the vendors. If no grouping information about the USP code was available from the USP, information from the vendors were used. At the moment, the list contains > 10,000 columns. It is available from our GitHub repository (https://github.com/michaelwitting/RtPredTrainingData/tree/master/resources/column_database) and is updated regularly with new columns. In order to collect a list of used solvents, additives and modifiers, all data descriptions obtained from MetaboLights and Metabolomics Workbench were searched for unique chemicals in the eluent composition. Standardized solvent and additive/modifier names were derived from ChEBI with their respective ChEBI ID and molecular formula. Based on manual literature mining, different abbreviations commonly used for solvents and additives have been collected.

## Results and discussion

### Collection of LC–MS metabolomics datasets

Since metadata of chromatographic separations is important for the (re-)use of RT data, we wanted to check how consistently it is provided across metabolomics data repositories. Therefore, we collected the content of the “Chromatography” subsection of 352 studies from MetaboLights (and additionally here the content of the “Assays” section) and 808 method descriptions of studies from the Metabolomics Workbench. If one study contained multiple described chromatographic separations, all of them were collected under the same MetaboLights or Metabolomics Workbench ID, but with our own internal ID. In total, 468 chromatographic descriptions from MetaboLights and 1033 from Metabolomics Workbench were collected and further analyzed. The notations of columns etc. were compared against the list of standardized column names, if possible. If no exact matching was possible due to ambiguities or if mistakes in the notation were made in any of the fields, this field was counted as complete, but obtained the flag “ambiguous/incorrect information”. We chose to study these two repositories since they contain a representative cross-section of fields in which metabolomics is applied. However, scientists submitting to these repositories value open science and data sharing and are typically aware of the importance of metadata. Therefore, the obtained data might overestimate the potential correctness of the chromatographic metadata provided elsewhere.

### Evaluation of completeness

**Fig. 1:**
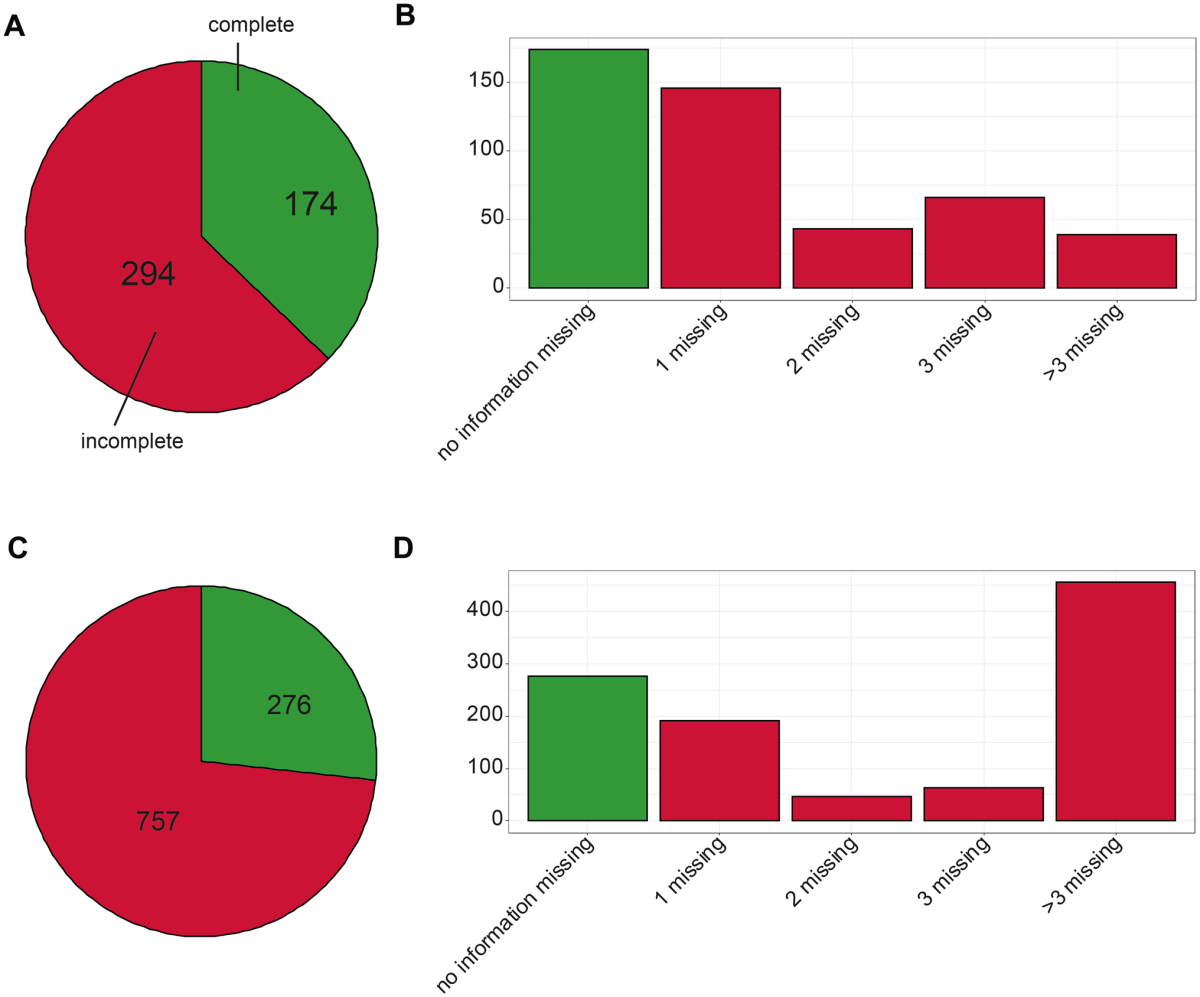
**A** Number of complete (green) and incomplete (red) datasets collected from EBI MetaboLights (in total 468 datasets). **B** Number of MetaboLights datasets, where no (green), one, two, three or more than three (red) piece of information are missing. **C** Number of complete (green) and incomplete (red) datasets collected from NIH Metabolomics Workbench (in total 1033 datasets). **D** Number of Metabolomics Workbench datasets, where no (green), one, two, three or more than three (red) piece of information are missing

In the first step, we evaluated how complete the descriptions of chromatographic systems were. The minimal information to be useful for further investigations was defined to consist of the column and its dimension (including inner diameter, length and particle size), the employed mobile phases, flow rate, column temperature and the programmed gradient and are similar to the MSI recommendations. Descriptions with no missing values (i.e. no “NA”) in any of the fields were considered to be complete. In the MetaboLights data this was the case for 174 (37.2%) descriptions, while in the remaining 294 (62.8%) descriptions information was missing for one or more of the fields (Fig. 1A). In the Metabolomics Workbench datasets, 276 (26.7%) descriptions were considered to be complete, while 757 (73.3%) descriptions were missing information in at least one of the fields (Fig. 1C).

In the second step, we examined more closely which fields were missing for the incomplete descriptions. In the MetaboLights data, 249 of the descriptions were missing the column temperature, followed by flow rate (127) and gradient program (113) (SI Fig. 1A). The Metabolomics Workbench data also frequently lack the column temperature (617), followed by the gradient (602) and flow rate (500) (SI Fig. 1B).

Third, if several fields were missing, we were interested which of them overlapped. In 146 MetaboLights descriptions, only a single field is missing, two fields are missing in 43, three in 66 and more than three in 39 descriptions (Fig. 1B). In the Metabolomics Workbench data, one field is missing in 192, two fields in 46, three in 63 and more than three in 456 descriptions (Fig. 1D). Of the latter, 355 descriptions in Metabolomics Workbench contain only information of the column (name, length, inner diameter and particle size).

We argue that for all descriptions with missing information the (re-)use of RT data might not be possible, because the employed chromatographic separation system cannot be correctly determined since important and essential information is not provided. For example, the temperature has a strong effect on the interaction of the analyte with the stationary and the mobile phase. Since changes in temperature do not affect each metabolite to the same extent, differences in RT and retention order might be observed when using different temperatures. Likewise, the choice of eluent has an effect on the RT and elution order since it influences the main separation principle (e.g. partitioning) and different secondary interactions (e.g. ionic interactions, hydrogen bonding), while the flow rate and the dimension of the column might not influence the retention order, but the absolute RT observed.

Initial analysis of the collected metadata showed that 1051 (70.0%) of all collected descriptions were incomplete. However, we found that even in the remaining 450 (30.0%) descriptions, which are regarded as complete, mistakes can be found or ambiguous annotations were used. These fields were counted as “ambiguous/incorrect information”. For selected examples, we observed insufficient or confusing notations of the column, the eluents or the gradient program, which will be discussed in the following paragraphs.

### Evaluation of ambiguous/incorrect annotations

In the case of chromatographic columns, the precise name and description of the column is required. For example, C_18_ columns from different vendors or even from a single vendor are not identical and differ from one another, e.g. in the base particle, carbon load or surface area, leading to differences in retentivity and selectivity. Thus, the stationary phases have different interactions with the metabolites due to diverse characteristics. Simply specifying “C_18_” or “C_8_″ is not sufficient to clearly identify the column. Furthermore, most of the columns are available with different lengths, inner diameters and particle sizes. These three parameters directly influence the absolute RT of metabolites, the separation efficiency and the width and shape of a peak. Stating the correct particle size is important, since some columns are available in different formats, e.g. as HPLC or U(H)PLC columns. One particular example is “Waters CORTECS” which is available with a 1.6 and 2.7 µm particle size. Both particle sizes are available in the same column formats, which can lead to an ambiguous column naming if the particle size is not explicitly stated, as columns with sub-2 µm particles are called U(H)PLC.

As there is often a variety of columns per manufacturer, the exact brand name and phase information are essential for a clear identification of the column. Certain notations do not uniquely identify a column, due to no or insufficient use of brand names. Selected examples are:

- “50 × 2.1 mm ACQUITY 1.7-µm C_18_ column (Waters Corp, Milford, MA)”, which could be a BEH, BEH Shield or HSS
- “ethylene-bridged hybrid (BEH) HILIC 2.1 × 150 mm, 1.7 µm; Waters”, which could be a ACQUITY or a XBridge
- “C-18 column (150 mm × 2.1 mm, 3.5 µm, Agilent, USA)”, which could be a ZORBAX, Polaris or Pursuit

In addition, the lack of exact phase information or the reporting of only the brand name also lead to ambiguous column notations. Detailed examples are:

- “Acclaim (Thermo Scientific) column, particle size 2.2 µm, 2.1 × 150 mm” might be an Acclaim 120 C_18_, HILIC or Mixed-Mode WAX-1
- “Luna, 3 µm particle size, 150 × 2 mm column (Phenomenex Macclesfield, Cheshire, UK)” might be a Luna C_18_, Omega C_18_ or Omega Polar C_18_

In the MetaboLights data, 174 datasets supposedly have complete data descriptions; yet, 10 of these datasets are ambiguous due to missing brand name or phase information. In the case of the Metabolomics Workbench data, the column information of 28 out of 276 datasets is insufficient. Additionally, we observed 32 and 40 data sets in MetaboLights and Metabolomics Workbench, respectively, where the explicit combination of column length/inner diameter/particle size, was not found in the list of standardized column names. In order not to miss we searched potential columns, column vendor brochures, websites and catalogs were searched, but no fitting columns were identified. In the future, our list with normalized column names can help prevent those “writing errors” in the future.

Besides the column, the eluents and their composition have the greatest influence on RT and retention order of the metabolites. We have observed inconsistencies for 4 and 12 descriptions in MetaboLights and Metabolomics Workbench, respectively. For example, in some cases the sum of the employed solvents added up to more than 100%. Detailed examples are:

- “65:35:5 Isopropanol/Methanol/Water (v/v/v) + 0.1% Formic Acid”
- “65:35:5 Isopropanol/Methanol/Water (v/v/v) + Ammonium Hydroxide”
- “MeOH/isopropanol/water (10:88:20 v/v/v) + 2 mM ammonium formate and 0.01% formic acid”

In other examples, the concentration or amount of some additives or solvents are not stated, e.g.:

- “90% ACN with pH 5.8 ammonium acetate”
- “ammonium carbonate and acetonitrile”
- “water-acetonitrile-formic acid”

The selected gradient conditions also strongly influence the RT. We found some inconsistencies in 9 of the MetaboLights and in 21 Metabolomics Workbench descriptions. We observed some gaps, overlaps or unspecified jumps in the gradient composition, e.g. “isocratic at 5% B (0–0.5 min), gradient from 10 to 75% B […]” or “[…] 98% B, 20–25 min; 27.5–37 min […]”. Moreover, we found some correct but hardly readable notations such as: “The gradient elution was as follows: global metabolomics; acetonitrile % = 3–3–60–75–100–100–3–3(0–0.5–4-6–6.1–8-8.1–10 min)”. Likewise, the next example seemed to be copied and inserted from a gradient table without formatting: “Time(min) Flow Rate(mL/min) %A %B Curve 1. Initial 0.400 99.0 1.0 2. 1.00 0.400 99.0 1.0 3. 16.00 0.400 1.0 99.0 4. 20.00 0.400 1.0 99.0 5. 20.50 0.400 99.0 1.0 6. 22.00 0.400 99.0 1.0”.

In summary, an additional 138 descriptions (31.7%) of the supposedly complete datasets do not allow further (re-)use of the information because either crucial information is missing, or the provided information contains errors or inconsistencies. In total, only 20.8% of all the collected descriptions (312 out of 1501 datasets) are complete and correct and can be used for further investigations.

### Best practice in reporting chromatographic metadata

(Meta-)Data representing chromatographic conditions in metabolomics repositories should be as self-explaining as possible, without the need to additionally track publications and should ideally be directly machine-readable. This would enable the direct (re-)use of RT data. Based on our observations, we suggest a list of minimal chromatographic information required in line with the recommendation of the MSI to enable the (re-)use of RT data. Different from the MSI recommendations, we additionally suggest a standardized nomenclature for eluent compositions, columns, gradients etc. Such standardization would enable automated data extraction and validation, e.g. upon deposition at repositories such as MetaboLights or Metabolomics Workbench. We exemplify the notation based on MTBLS291 and compare the current method description with the new notation (see supplementary information).

While we did not explicitly check for the used LC instrumentation, stating this information can help in identifying some specific peculiarities with the RT data, e.g. dead volume size or quality of the data. HPLC systems typically have higher dead volumes compared to modern U(H)PLC systems and certain separations might be performed on both systems. The complete model name of the instrument and all potentially present sub modules shall be stated, e.g. additional UV-detectors in line increase the dead volume after the column. Any larger modifications, e.g. addition of flow splitting systems etc., shall be specified to get a better idea of the quality of RT data.

### Column

Most importantly the chromatographic column needs to be correctly stated. This includes the manufacturer, model name, column inner diameter, column length and particle size. If a guard column is used, the same applies to the guard column. Exact names can be retrieved from catalogs of vendors and the packaging. We have collected a list of currently more than 10,000 columns specifications, including manufacturer name, brand name, column dimensions, particle size and pore size together with the USP code (see Sect. 2). This list is freely available and updated on a regular basis (https://github.com/michaelwitting/RtPredTrainingData/tree/master/resources/column_database). It may serve as a white list for reviewers and data curators who want to know if certain columns exist, although it should be noted that this list is not complete and contains mainly known manufacturers and “normal” and regularly used columns. The exact column name should be followed by the dimensions of the column in brackets using mm as unit of measure, e.g. “150 mm × 2.1 mm ID”. In case of micro- and nanoflow columns the inner diameter can be also written in µm according to current conventions, for example “150 mm × 300 µm ID” or “150 mm × 0.3 mm ID”. Since several column chemistries span the entire range of particle sizes, it is important to specify this as well, e.g. “1.6 µm”. One example for a complete column description is “Waters CORTECS UPLC C_18_ (150 mm × 2.1 mm ID, 1.6 µm)”. Sometimes columns with different pore sizes are available, if this is the case, it should be added after the particle size, e.g. “Waters CORTECS UPLC C_18_ (150 mm × 2.1 mm ID, 1.6 µm, 90Å)”.

### Eluents

At present, there are no specific rules on how to report the composition of eluents, which means that each user describes the composition of the solvent in his/her own style. The order of the solvents and additives, the used abbreviations and the relative composition are written in many different ways, which makes automatic retrieval and comparison complicated. For eluents, two cases need to be considered. In the first case the final composition of the eluent is described with all final percentages and concentrations (e.g. “10% H_2_O/90% ACN + 10 mM ammonium formate”). The second case describes a recipe for how to prepare an eluent (e.g. “90% ACN + 10% 100 mM ammonium formate”). The two examples just given in brackets result in the same solvent, but with different notations. In a detailed protocol, both should be reported. Furthermore, the name, source and purity of each component should be indicated, typically in the material and methods section or the supporting information of a publication but also in repositories. Concentrations, masses and volumes to prepare a given concentration need to be defined as v/v, v/w or w/w. When describing an eluent, the final composition using the standard solvent abbreviations as summarized in Table 1 should be used. However, it is suggested to expand the protocol sections by how exactly the eluents have been prepared (A was added to B or B was added to A, etc.). Hereby, the used solvents shall be ordered based on their eluotropic strength in RP chromatography, since it is the currently most commonly used separation method in metabolomics. Ideally, mixtures are reported using volume fraction (v/v) in % or reported as such with adding “(v/v)”. If other measures (e.g. mass fraction) have been used, this needs to be indicated and solvents should add up to 100%. Individual solvents are separated by a “/”, e.g. “10% H_2_O / 90% ACN”. The solvent composition is followed by all additives in alphabetic order and separated by a “ + ”. If possible, full names of additives should be used to avoid confusion, e.g. “10% H_2_O / 90% ACN + 10 mM ammonium formate / 0.1% formic acid”. A particular example is the case of ammonium formate and ammonium fluoride which both might be abbreviated with “AmF” or “NH_4_F”. Commonly used additives, with their full names, ChEBI identifiers and formulas are summarized in Table 2.

**Table 1:** Common LC solvents and their abbreviations

Name	ChEBI ID	Formula	Abbreviation(s)
Acetone	CHEBI:15347	C_3_H_6_O	ACE
Acetonitrile	CHEBI:38472	C_2_H_3_N	ACN, MeCN
1-Butanol	CHEBI:28885	C_4_H_10_O	nBuOH, BuOH
Chloroform	CHEBI:35255	CHCl_3_	CHCl_3_
Cyclohexane	CHEBI:29005	C_6_H_12_	Cy, Cyhex
Dimethyl formamide	CHEBI:17741	C_3_H_7_NO	DMF
Dimethylsulfoxide	CHEBI:28262	C_2_H_6_OS	DMSO
1,4-Dioxane	CHEBI:47032	C_4_H_8_O_2_	
Ethyl acetate	CHEBI:27750	C_4_H_8_O_2_	EtOAc
n-Heptane	CHEBI:43098	C_7_H_16_	
n-Hexane	CHEBI:29021	C_6_H_14_	
Isooctane	CHEBI:62805	C_8_H_18_	
Methanol	CHEBI:17790	CH_4_O	MeOH
Methyl-tert-butyl ether	CHEBI:27642	C_5_H_12_O	MTBE
Methylethyl ketone	CHEBI:28398	C_4_H_8_O	
Dichloromethane	CHEBI:15767	CH_2_Cl_2_	DCM
2-Propanol	CHEBI:17824	C_3_H_8_O	iPrOH
1-Propanol	CHEBI:28831	C_3_H_8_O	nPrOH
Tetrahydrofuran	CHEBI:26911	C_4_H_8_O	THF
Toluene	CHEBI:17578	C_7_H_8_	
Water	CHEBI:15377	H_2_O	H_2_O

**Table 2:** Common additives used in metabolomics

Name	ChEBI ID	Formula
Formic acid	CHEBI:30751	CH_2_O_2_
Acetic acid	CHEBI:15366	C_2_H_4_O_2_
Ammonium acetate	CHEBI:62947	C_2_H_7_NO_2_
Ammonium formate	CHEBI:63050	CH_5_NO_2_
Ammonium carbonate		CH_8_N_2_O_3_
Perfluoropentanoic acid	CHEBI:83491	C_5_HF_9_O_2_
Ammonium bicarbonate		CH_5_NO_3_
Ammonium fluoride	CHEBI:66871	FH_4_N
Ammonium hydroxide	CHEBI:18219	H_5_NO
Ammonia	CHEBI:16134	H_3_N
N,N-Dimethylhexylamine		C_8_H_19_N
Phosphoric acid	CHEBI:26078	H_3_O_4_P
Tributylamine	CHEBI:38905	C_12_H_27_N

An important factor in preparation of eluents is the adjustment of pH. The commonly used pH-scale is valid for aqueous solutions. Typically, the pH is adjusted in the aqueous part before mixing with organic solvents. However, mixing with an organic solvent leads to shifts in the pH due to changes in proton activity. pH values measured in hydro-organic mixtures should be reported as apparent pH*, e.g. “5% H_2_O / 95% ACN + 10 mM ammonium acetate, pH* 4.6”. In such cases it is important to report exactly when and in which solvent the pH was adjusted, which acid or base was used and how the different ingredients were mixed.

### Column temperature and flow rate

Column temperature influences the mobile phase temperature, the viscosity (and therefore back pressure) and the selectivity of a separation since changes in temperature do not affect different metabolites to the same extent. While an increase in temperature generally decreases retention time, a change in elution order might be observed for certain metabolite pairs. Precise statements about the temperature at which the separation was carried out is required. The same is true for the flow rate. Typically, separations are carried out at a constant flow rate. However, flow rate might be adjusted during gradients for improved performance or for flushing and re-equilibration of the column. In case of one single flow rate, it is recommended to be specified in mL/min, µL/min or nL/min (or mL·min^−1^, µL·min^−1^ or nL·min^−1^), whatever is most appropriate. In case of multiple flow rates, they have to be reported together with the gradient indicating the time point of the change/variation.

### Gradient

Gradients are ideally presented in the form of a table, containing exact time, percentages of eluents A, B, C, D, the gradient curve (note that this might be different for different instrument vendors, therefore the instrument becomes important to report) and if flow rates change over time, then the correct flow rate at the specified time point.

Since gradient programming in LC control software refers to mixtures of the different eluents and not the amount of solvent, the description of a gradient should also depict this. Descriptions like “… the ACN content was increased to 90% at 15 min …” should be avoided, since it is not clear if 90% is referring to the percentage of the solvent or the mixing of the eluents. Furthermore, it is generally easier to reproduce gradients if the specific time points of changes are indicated instead of durations (e.g. “… a linear increase for 10 min …”). If the description in the form of a table is not possible, we propose to use a notation following the gradient description in MassBank records. Here the relative proportions of the eluents at a specific time point are separated by “/”, e.g. “10/90 at 1 min”. Different time points are separated by a comma. The complete analytical gradient shall be described including re-equilibration times. Lastly, if data acquisition was only performed during a specific time frame or for example the effluent of the LC was diverted to waste instead of the MS, these times need to be reported. In case of changes of the flow rate in the gradient this has to be indicated in a similar way, e.g. “40/60 at 0.1 mL/min at 5 min”.

### Use of regular expressions to retrieve information

**Fig. 2:**
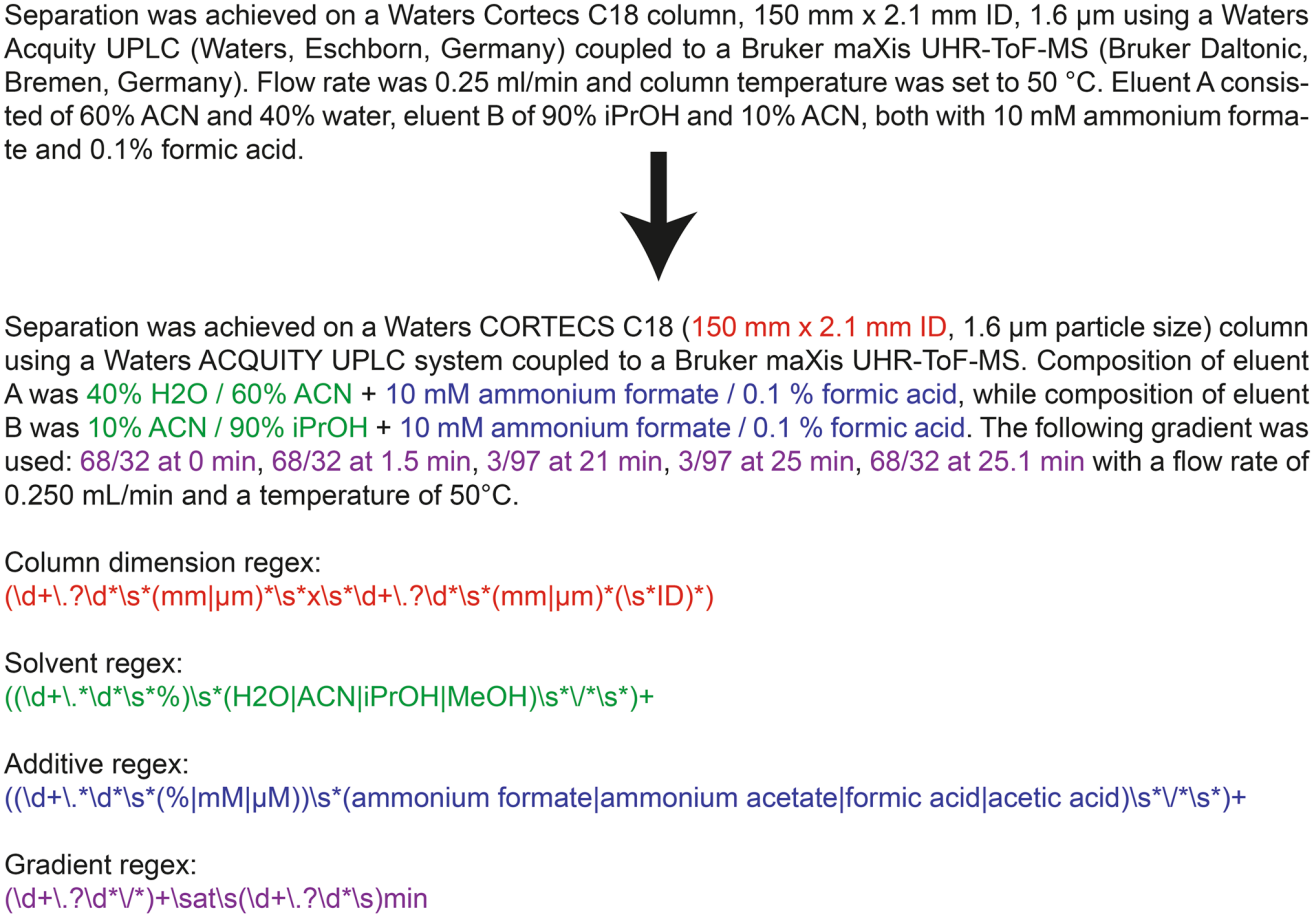
Correct and complete notation using the example of study MTBLS291 from MetaboLights with additional isolation of the text blocks on the column, eluents and additives and the gradient using regular expressions via regexr.com

To further illustrate the usability of the new notation we generated examples of regular expressions for isolating specific information from a text block like above. The examples are found in Fig. 2 and the regexr.com website. The first example illustrates how the final solvent composition from the eluents can be retrieved (regexr.com/59lia) as well as the additives (regexr.com/59lid). The second example shows how the gradient information is isolated (regexr.com/59lig), while the last isolates column dimensions (regexr.com/59lij). Figure 2 shows the regular expressions and the matched text blocks. Similar regular expressions or search patterns can be created for column names using the list from our GitHub repository (https://github.com/michaelwitting/RtPredTrainingData/tree/master/resources/column_database).

## Conclusion

Sharing and (re-)use of any kind of data heavily relies on the quantity and quality of data and metadata. While for sharing of MS data, this is well-established and understood, collection and sharing of RT data is not as widespread. In this study we analyzed publicly available data descriptions of LC–MS based metabolomics studies retrieved from the EBI MetaboLights and NIH Metabolomics Workbench repositories. Since experimental descriptions in these repositories are often taken from accompanying publications, they are a good proxy for the current practice of describing chromatographic methods. We first evaluated the completeness of data descriptions using 8 different properties: column name, column dimension (length, inner diameter and particle size), eluent descriptions, temperature, flow rate and gradient. From 468 investigated chromatographic descriptions at MetaboLights and 1033 descriptions at Metabolomics Workbench, 146 or 192, respectively, were missing one piece of information, whereas in 148 or 565 respectively, even two or more were missing. Furthermore, supposedly complete datasets have several weaknesses in their description, e.g. by incorrect or incomplete column naming, eluent description etc. In total, only about 20% of the datasets provide all required information to facilitate a simple and comprehensive (re-)use of the RT data.

Based on these observations, we suggest a more standardized system for the description of chromatographic separation conditions. Standardized ways of writing and describing of such a system allows generating automatic checks, e.g. for data submission to public repositories, but also enables extracting information in an automated manner for (re-)use in other contexts. We supply suggestions for the correct naming of columns, solvents, additives and gradients. However, upload of metadata should be made easy for authors, who wish to share their data with the community. Automatic extraction of information, e.g. from.mzML files, represents the best option. This would require that information is captured in the data acquisition software. This is often the case for column temperature, flow rate, gradient and instrument model, but the exact column and used eluents are often not part of this. The support of column and instrument vendors will be required to enable this. In the future, more detailed and accurate descriptions will allow using RT information across different systems, such as PredRet or other approaches, in a more detailed and more accurate way (Stanstrup et al., 2015; Vaughan et al., 2012). This will improve the way how RT is used in metabolite identification and for the development of new computational tools.

## Supplementary Information

Below is the link to the electronic supplementary material.

Supplementary file1 (DOCX 14 kb)

Supplementary file2 (TIF 3896 kb)

Supplementary file3 (XLSX 207 kb)

## Data Availability

Curated data is available from GitHub (https://github.com/michaelwitting/RtPredTrainingData/tree/master/resources/column_database) and the Supplementary Information.
