# Supplementary material for: Critical assessment of chromatographic metadata in publicly available metabolomics data repositories
Source: Metabolomics. 2022 Nov 27;18(12):97. doi: 10.1007/s11306-022-01956-x (PMC9701651; doi:10.1007/s11306-022-01956-x)
Supplement: Supplementary file 1 — Supplementary file1 (DOCX 14 kb) [file 11306_2022_1956_MOESM1_ESM.docx]

**Critical assessment of chromatographic metadata in publicly available metabolomics data repositories – Supplementary Information**

Eva-Maria Harrieder^1^, Fleming Kretschmer^2^, Warwick Dunn^3^, Sebastian Böcker^2^ and Michael Witting^4,5,^*

^1^ Research Unit Analytical BioGeoChemistry, Helmholtz Zentrum München, Ingolstädter Landstraße 1, 85764 Neuherberg, Germany

^2^ Chair of Bioinformatics, Friedrich-Schiller-Universität Jena, Ernst-Abbe-Platz 2, 07743 Jena, Germany

^3^ Department of Biochemistry and Systems Biology, Institute of Systems, Molecular, and Integrative Biology, University of Liverpool, Liverpool L69 7ZB, UK

^4^ Metabolomics and Proteomics Core, Helmholtz Zentrum München, Ingolstädter Landstraße 1, 85764 Neuherberg, Germany

^5^ Chair of Analytical Food Chemistry, TUM School of Life Sciences, Technical University of Munich, Maximus-von-Imhof-Forum 2, 85354 Freising, Germany

* corresponding author

Dr. Michael Witting

michael.witting@helmholtz-muenchen.de

*Case study - MTBLS291*

We would like to illustrate the application of the suggested notation on the example of MetaboLights study MTBLS291. The current method description reads as followed: “Separation was achieved on a Waters Cortecs C_18_ column, 150 mm x 2.1 mm ID, 1.6 µm using a Waters Acquity UPLC (Waters, Eschborn, Germany) coupled to a Bruker maXis UHR-ToF-MS (Bruker Daltonic, Bremen, Germany). Flow rate was 0.25 ml/min and column temperature were set to 50°C. Eluent A consisted of 60% ACN and 40% water, eluent B of 90% iPrOH and 10% ACN, both with 10 mM ammonium formate and 0.1% formic acid.”

The column name is almost correct, the particle size indicates a sub-2-µm particle. Therefore, the column name according to our curated list together with column dimensions will be “Waters CORTECS UPLC C_18_ (150 mm x 2.1 mm ID, 1.6 µm, 90Å)”. While eluent composition could be readily retrieved, it can be improved by applying the notation suggested in this publication. The compositions of the eluents will be written as solvent A is “40% H_2_O / 60% ACN + 10 mM ammonium formate / 0.1% formic acid” and solvent B will be “10% ACN / 90% iPrOH + 10 mM ammonium formate / 0.1% formic acid”. No gradient was indicated, searching in the original publication describing the method the gradients should be written as follows: “68/32 at 0 min, 68/32 at 1.5 min, 3/97 at 21 min, 3/97 at 25 min, 68/32 at 25.1 min”. Furthermore, in this description no details on the preparation of the mobile phases or the gradient curve have been mentioned. The final description should read as follows: “Separation was achieved on a Waters CORTECS C_18_ (150 mm x 2.1 mm ID, 1.6 µm, 90Å) column using a Waters ACQUITY UPLC system coupled to a Bruker maXis UHR-ToF-MS. Composition of eluent A was 40% H_2_O / 60% ACN + 10 mM ammonium formate / 0.1% formic acid, while composition of eluent B was 10% ACN / 90% iPrOH + 10 mM ammonium formate / 0.1% formic acid. The following linear gradient was used: 68/32 at 0 min, 68/32 at 1.5 min, 3/97 at 21 min, 3/97 at 25 min, 68/32 at 25.1 min with a flow rate of 0.250 mL/min and a temperature of 50°C.” In this case linear gradient refers to the gradient curve since different settings in different vendor software have different meaning. However, in most cases a linear gradient program is used in metabolomics. The additional eluent preparation could read like this: “For preparation of eluent A 400 mL of H_2_O were mixed with 600 mL of ACN followed by the addition of 1 mL 10 M ammonium formate solution and 1 mL formic acid. Likewise, eluent B was prepared by mixing 100 mL ACN and 900 mL iPrOH followed by the addition of 1 mL 10 M ammonium formate and 1 mL formic acid. Since especially in eluent B ammonium formate might initially precipitate, both eluents were sonicated until ammonium formate dissolved completely.”
