# Supplementary figures and images for: Critical assessment of chromatographic metadata in publicly available metabolomics data repositories
Source: Metabolomics. 2022 Nov 27;18(12):97. doi: 10.1007/s11306-022-01956-x (PMC9701651; doi:10.1007/s11306-022-01956-x)

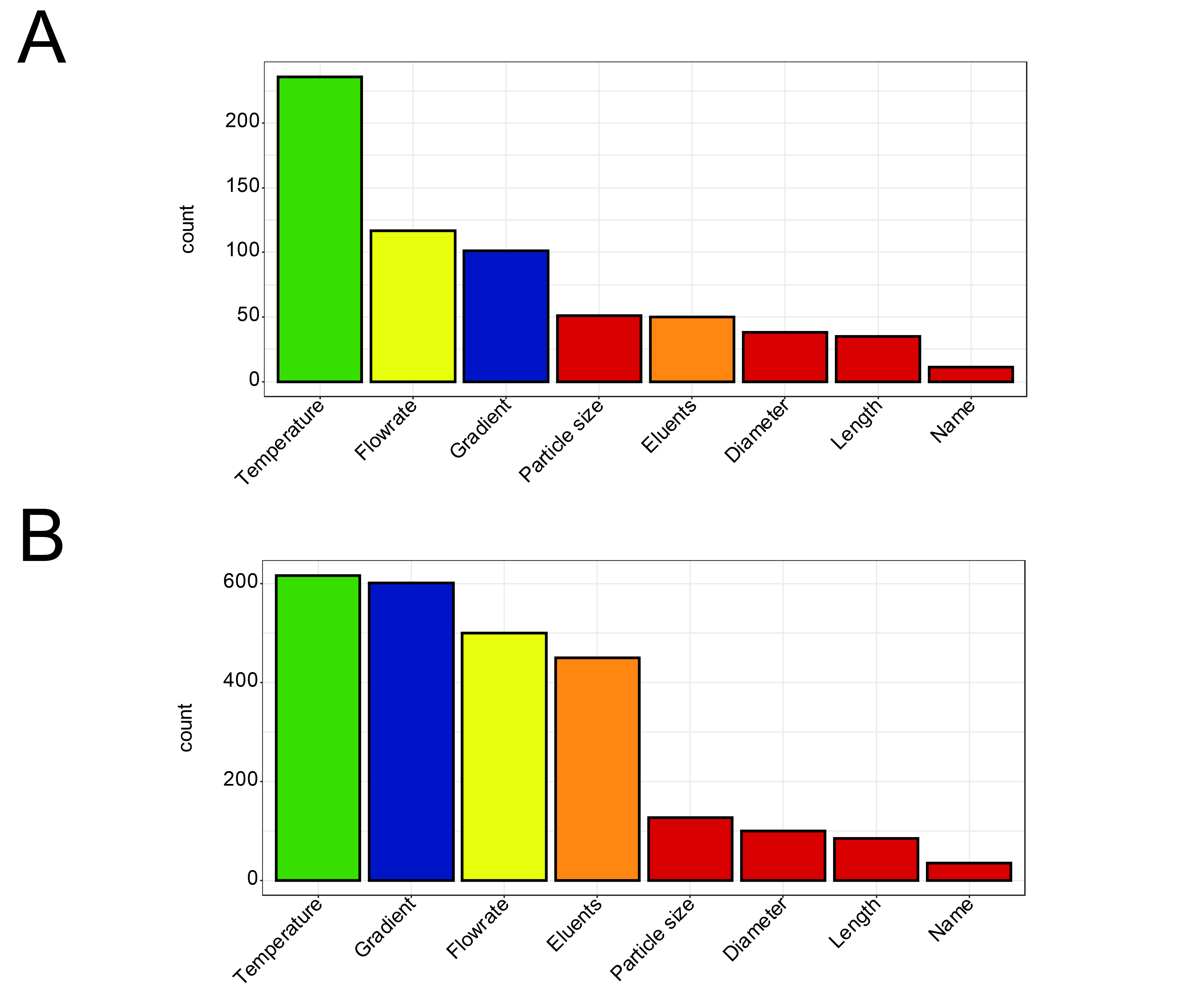

Supplement: Supplementary file 2 — Supplementary file2 (TIF 3896 kb) [file 11306_2022_1956_MOESM2_ESM.tif]
